# Metformin-associated Lactic Acidosis: An Unexpected Scenario

**DOI:** 10.7759/cureus.4397

**Published:** 2019-04-05

**Authors:** Samia Asif, Joseph Bennett, Bindiya Marakkath

**Affiliations:** 1 Internal Medicine, University of Missouri Kansas City (UMKC), Kansas City, USA; 2 Internal Medicine, University of Missouri Kansas City (UMKC), Kansas city, USA

**Keywords:** metformin, lactic acidosis, metformin toxicity

## Abstract

According to the National Diabetes Statistics Report (2017) by Centers for Disease Control and Prevention (CDC), 9.4% of the US population, approximately 30.3 million people had diabetes while 84.1 million had pre-diabetes as of 2015. In addition to lifestyle changes, the American Diabetes Association recommends metformin as the first-line treatment for type 2 diabetes. Hence, not surprisingly, metformin is a commonly prescribed medication by most healthcare providers in all clinical settings. As a result, it remains essential that all medical professionals be aware of any adverse effects as a result of metformin therapy, no matter how uncommon.

We present the case of a 42-year-old lady with type 2 diabetes mellitus who required initial admission to intensive care unit (ICU) after presenting with unilateral back and lower abdominal pain with dysuria and was noted to have an acute kidney injury with a creatinine of 7.45 mg/dL and severe metabolic acidosis with a pH of 6.7 and an anion gap more than 50 mmol/L. Lactic acid was elevated at 24.2 mmol/L. Serum metformin levels were high at 14 mcg/mL (therapeutic range: 1-2 mcg/mL). She required emergent dialysis but subsequently, renal functions recovered.

Risk of metformin-associated lactic acidosis (MALA) is reported to be an estimated 6.3 per 100,000 patient-years. Commonly encountered clinical scenarios such as hypoxemia, sepsis, alcohol abuse, renal injury, and shock can precipitate MALA. Early recognition allows timely initiation of appropriate therapy and reduces associated morbidity.

## Introduction

Metformin is currently recommended as a first-line agent in the management of type 2 diabetes in association with lifestyle modifications by the American Diabetes Association. It is considered cost effective as well as safe given lack of hypoglycemia as a side effect. Commonly reported adverse effects include gastrointestinal symptoms such as diarrhea and abdominal cramps; lactic acidosis remains a recognized but rare complication. An estimated incidence of metformin-induced lactic acidosis (MALA) is 6.3 per 100,000 patient-years [1]. Mortality rate is reported at 50% [2]. A comprehensive literature search performed by Salpeter et al. to compare incidence of lactic acidosis in patients treated with metformin compared to nonmetformin therapies or placebos which included 347 trials and cohort studies suggested no evidence that metformin was associated with increased risk of lactic acidosis [3]; however, multiple cases of MALA are reported, suggesting the inability of controlled trials to reveal this complication may be because in all these meticulous studies, metformin is prescribed appropriately, keeping in mind recommended contraindications and in patients who lack risk factors that predispose to MALA, something that is not always the case in true clinical practice. Here, we report the case of a 42-year-old lady who presented with acute kidney injury in setting of hypovolemia, with severe metabolic acidosis secondary to lactic acidosis and markedly elevated metformin levels.

## Case presentation

A 42-year-old Caucasian female with past medical history significant for type 2 diabetes mellitus with gastroparesis, asthma, hypertension, and coronary artery disease presented to the emergency room (ER) with complaints of acute onset right-sided back pain in association with lower abdominal pain and dysuria ongoing for the last one week. She also reported having nonbloody, watery diarrhea for the last one month and multiple episodes of nonbloody, nonbilious vomiting over the same time. She denied any fevers. In the ER, patient was noted to be markedly hypertensive with a blood pressure of 207/148 mmHg on admission. She was afebrile and oxygen saturation was 100% at room air. The patient was tachypneic with a respiratory rate of 26 breaths/minute. Right costovertebral angle tenderness as well as lower abdominal tenderness was noted. She reported good compliance to her home medications which included lisinopril 40 mg daily and metformin 1000 mg twice daily. 

Initial laboratory results showed high anion gap metabolic acidosis with an arterial blood gas showing a pH of 6.7 (normal range: 7.35-7.45), partial pressure of carbon dioxide (PaCO2) of 16.2 mmHg (normal range: 35.0-45.0 mmHg), and bicarbonate levels were reported as less than 2 mEq/L (normal range: 22-28 mEq/L). Anion gap was reported as more than 50 mmol/L. Her creatinine was elevated at 7.45 mg/dL with a BUN 54 mg/dL. The patient did not have a known history of any kidney impairment. Her blood glucose levels were 96 mg/dL, serum osmolality was 311 mOsm/kg, and lactic acid was elevated at 24.2 mmol/L. Blood alcohol and salicylate levels were normal. Creatinine kinase was 160 units/L, within a normal range for our facility. Given elevated blood pressure at presentation and acute back pain, computed tomography (CT) angiography of chest, abdomen, and pelvis for possible dissection was immediately obtained, inspite of the impaired renal function. No significant abnormality on chest, abdominal, and pelvic imaging was reported. The patient was noted to have a white cell count of 18,000/mm³ with 63% neutrophils; hemoglobin on presentation was 15.1 g/dL with an unknown baseline and a platelet count of 497,000/mm³. Urine drug screen was negative. 

Once patient received intravenous morphine (single dose of 4 mg) for pain control, her blood pressure dropped to 104/68 mmHg. She received two liters of lactated Ringer's while in the ER. She continued to remain tachypneic with a respiratory rate of 30 breaths/minute. Nephrology was consulted; sodium bicarbonate infusion was started. She was transferred to the ICU where decision to proceed with emergent hemodialysis was made. Blood, urine, and sputum cultures were obtained. The patient was empirically started on vancomycin and piperacillin/tazobactam. As the patient became more hypotensive, inotropic support with norepinephrine infusion was started. Dialysis was initiated and the patient underwent a single session without ultrafiltration via a temporary right internal jugular vein dialysis catheter. She denied any use of nonsteroidal anti-inflammatory drugs (NSAIDs). As per nephrology, etiology of acute kidney injury was felt to be prerenal in setting of volume depletion due to reported vomiting and diarrhea and poor oral intake. Her severe metabolic acidosis with high anion gap was felt to be secondary to lactic acidosis due to metformin toxicity in setting of acute renal failure and hemodialysis was expected to clear the metformin.

After a single session of hemodialysis, her metabolic acidosis resolved. By the time of discharge from the hospital, her creatinine had recovered to 1.13 mg/dL with a bicarbonate level of 26 mEq/L. Her white cell counts normalized and her hemoglobin was subsequently noted to be stable at 9.6 g/dL. She became hemodynamically stable, no longer requiring inotropic support. Metformin levels were checked; this was a laboratory test sent out to another facility and hence results were not available immediately; these were later reported to be as high as 14 mcg/mL with the therapeutic range being 1-2 mcg/mL. Antibiotics were discontinued as blood and urine cultures remained negative. She was transferred out of the ICU and was subsequently discharged home in stable condition.

## Discussion

Metformin is a commonly used oral anti-hyperglycemic agent, frequently used as a first-line agent by providers. It is considered a safe medication with most commonly reported side effects being related to the gastrointestinal tract, such as diarrhea and nausea. Due to risk of causing lactic acidosis, its use is contraindicated if there is underlying renal or hepatic insufficiency. Risk of metformin-induced lactic acidosis is reported to be low with reported incidence less than 10 events per 100,000 patient-years of exposure [4].

Metformin belongs to the biguanide group of anti-hyperglycemic agents. Its mechanism of action involves increasing peripheral uptake of glucose while decreasing gluconeogenesis and fatty acid oxidation. Metformin-associated lactic acidosis (MALA) is characterized by a blood pH level less than 7.35 and an arterial blood lactate level more than 5 mmol/L [5]. It leads to increased levels of lactate by inhibiting lactate removal in liver and muscles at level of mitochondrial respiratory chain complex where lactate is oxidatively metabolized. With therapeutic doses of metformin, plasma lactate concentrations are usually less than 2 mmol/L. It is renally excreted in unchanged form. As per current guidelines, metformin should not be used if glomerular filtration rate (GFR) is less than 30 mL/min/1.73 m^2^ and dose to be reduced and adjusted when GFR is less than 45 mL/min/1.73 m^2^ [6].

**Figure 1:**
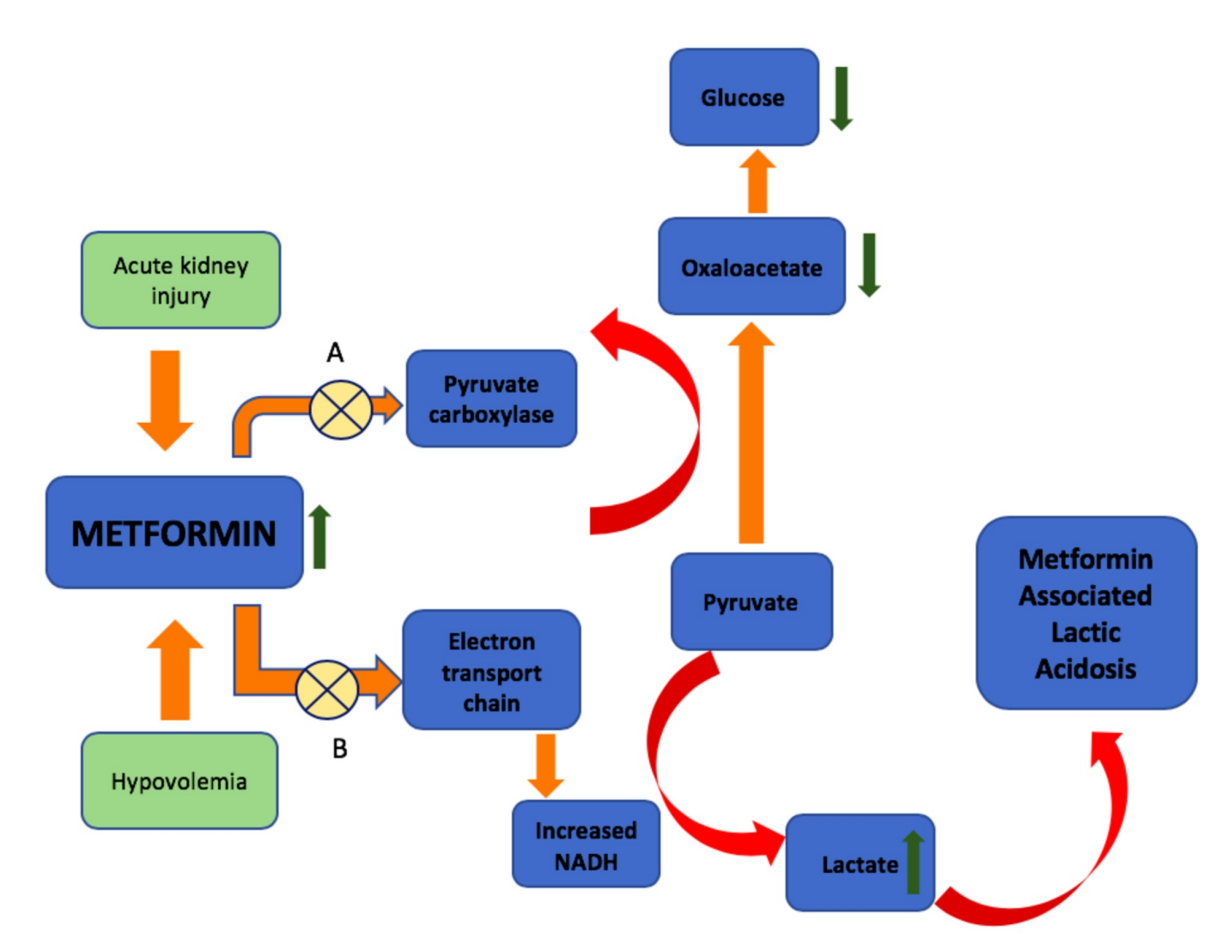
Development of metformin-associated lactic acidosis (MALA) in setting of hypovolemia and acute kidney injury; elevated metformin levels result in (A) inhibition of enzymes involved in gluconeogenesis, reducing conversion of pyruvate to glucose and (B) inhibiting mitochondrial electron transport chain, resulting in increased conversion of pyruvate to lactate. NADH, reduced nicotinamide adenine dinucleotide.

Development of MALA involves a combination of lactate overproduction and drug accumulation, such as seen with poor tissue perfusion in setting of hypovolemic shock and concurrent chronic or acute renal impairment. Within the hepatocytes, metformin inhibits gluconeogenesis, reducing production of glucose from pyruvate via the Krebs cycle and resulting in conversion of pyruvate to lactate [7]. In setting of hypoxia or tissue hypo-perfusion, metformin itself can inhibit mitochondrial oxidative phosphorylation through the electron transport chain; this decreases synthesis of adenosine triphosphate (ATP) and increases the amount of reduced nicotinamide adenine dinucleotide (NADH); ultimately this results in increased amounts of pyruvate that is also converted to lactate [8]. This is summarized in Figure 1.

Risk factors predisposing to MALA are common, such as hypoxemia, sepsis, alcohol abuse, renal injury, and shock [9]. It is most frequently seen in patients, such as in the reported case, when a patient develops an acute renal injury particularly in setting of volume depletion such as with diarrhea and vomiting. Levels of metformin are more than 5 mcg/mL [9] when lactic acidosis is attributed to metformin. Medications such as angiotensin converting enzyme inhibitors and angiotensin receptor blockers that affect auto-regulation within the kidneys and gastrointestinal volume losses are noted to cause acute renal impairment that produces MALA [10]. Of note, metformin plasma level measurement is not available in all laboratories which may delay diagnosis if not clinically suspected. Current first-line treatment remains utilizing renal replacement therapies such as hemodialysis or continuous renal replacement therapy if hemodynamic stability is a concern to remove metformin from the blood stream. Sodium bicarbonate can be given to correct associated metabolic acidosis. Concurrent insults such as hypovolemia need to be corrected as well [11].

## Conclusions

As medical practitioners, patients with type 2 diabetes are encountered on a daily basis and metformin is commonly prescribed. MALA is a rare and hence, less expected complication of metformin therapy but one with higher mortality rate; making it essential that all practitioners are aware of it. Timely diagnosis allows correct treatment that is lifesaving. Simple measures such as correctly prescribing metformin and respecting contraindications are preventative.
